# Classification of M1/M2-polarized human macrophages by label-free hyperspectral reflectance confocal microscopy and multivariate analysis

**DOI:** 10.1038/s41598-017-08121-8

**Published:** 2017-08-21

**Authors:** Francesca R. Bertani, Pamela Mozetic, Marco Fioramonti, Michele Iuliani, Giulia Ribelli, Francesco Pantano, Daniele Santini, Giuseppe Tonini, Marcella Trombetta, Luca Businaro, Stefano Selci, Alberto Rainer

**Affiliations:** 1 Institute for Complex Systems, National Research Council, via del Fosso del Cavaliere 100, 00133 Rome, Italy; 2 Microfabrication group, Institute for Photonics and Nanotechnology, National Research Council, via Cineto Romano 42, 00128 Rome, Italy; 3 Tissue Engineering Unit, Università Campus Bio-Medico di Roma, via Álvaro del Portillo 21, 00128 Rome, Italy; 4 Translational Oncology Unit, Università Campus Bio-Medico di Roma, via Álvaro del Portillo 21, 00128 Rome, Italy; 5 IFN/UCBM Joint Lab for Nanotechnologies for the Life Sciences (nano4life), Rome, Italy

## Abstract

The possibility of detecting and classifying living cells in a label-free and non-invasive manner holds significant theranostic potential. In this work, Hyperspectral Imaging (HSI) has been successfully applied to the analysis of macrophagic polarization, given its central role in several pathological settings, including the regulation of tumour microenvironment. Human monocyte derived macrophages have been investigated using hyperspectral reflectance confocal microscopy, and hyperspectral datasets have been analysed in terms of M1 vs. M2 polarization by Principal Components Analysis (PCA). Following PCA, Linear Discriminant Analysis has been implemented for semi-automatic classification of macrophagic polarization from HSI data. Our results confirm the possibility to perform single-cell-level *in vitro* classification of M1 vs. M2 macrophages in a non-invasive and label-free manner with a high accuracy (above 98% for cells deriving from the same donor), supporting the idea of applying the technique to the study of complex interacting cellular systems, such in the case of tumour-immunity *in vitro* models.

## Introduction

Besides their involvement in several physiological and pathological processes^1, 2^, macrophages have been identified as key players in orchestrating the tumour microenvironment^3, 4^. Tumour-Associated Macrophages (TAMs) are indeed a major cellular component of cancer-related inflammation, which can exert dual influence on cancer depending on their activation, with classically (M1) and alternatively (M2) polarized macrophages exhibiting anti-tumoural and pro-tumoural functions, respectively^5^. TAMs have been shown to affect several aspects of tumours, including: (i) self-renewal and drug resistance of cancer stem cells^6^; (ii) promotion of tumour-associated angiogenesis^7^; (iii) invasion^8^ and metastasis^9^; and (iv) suppression of antitumor immune response^10^. In accordance with these findings, TAMs are therefore emerging as a potential clinical target of cancer immunotherapy strategies^11, 12^, and clinical studies have recently been directed toward the characterization of the role of TAMs in disease. Assessment of TAM polarization via genetic characterization (expression of 12 single-nucleotide polymorphisms in 8 genes involved in TAM polarization) has been evaluated in metastatic colorectal cancer patients^13^, revealing a significant association with the clinical outcome. In another study, the association of M2 polarized cells with decreased sensitivity of EGFR treatment in patients with lung adenocarcinoma has been observed^14^. Besides clinical studies, the development of advanced *in vitro* models to gather relevant data on the involvement of macrophages in orchestrating the tumour microenvironment is also gaining increasing attention^15, 16^.

In such a scenario, the possibility of monitoring and discriminating single cells without perturbing their activation characteristics and time evolution has an enormous theranostic potential. The chance of gaining information from cells living and evolving in their environment opens new perspectives in drug testing, cell therapy, regenerative medicine, personalized immunotherapy and cancer treatment. Therefore, the definition of new methods for label-free and non-invasive high resolution imaging represents one of the main goals of several methodological and instrumental approaches.

An example is represented by the recent evolution of nonlinear microscopy^17–19^, which has been accompanied by the development of high-power tunable lasers, optimized objectives and high-sensitivity photomultipliers with a wide dynamic range. These technological improvements have recently enabled the application of Third Harmonic Generation (THG) microscopy to three-dimensional (3D) cell and tissue specimens, complementing Two-Photon Excitation Fluorescence (TPEF) and Second Harmonic Generation (SHG) detection^20^ in the 3D visualization of extracellular matrix structures, cell morphology and subcellular organization for multi-parameter analysis of cell–tissue interaction and function.

Nonlinear optical microscopy has been successfully applied to translational research, demonstrating its potential in evidencing alterations in collagen orientation and density within neoplastic tissues^21, 22^, as well as in the detection of autofluorescence signals from intracellular components which can reliably be associated to pathological conditions^23^.

Remarkably, imaging methods are going toward the definition of image-based systems biology approaches^24, 25^ which may better represent a “global analysis” of cell features, from morphology to the overall biochemical dynamic equilibrium, rather than seeking specific markers of a uniquely identified cell state.

Several cell properties change with specific lineage, differentiation step or evolving disease. Some of these changes, for example in the nuclear structure, in the spatial distribution and metabolic activity of mitochondria, or in the spatial distribution and overall expression of cytoskeletal proteins, can be monitored non-invasively for phenotype and functional characterization. To this end, several spectral approaches have been developed and are evolving, using different spectral ranges and molecular fingerprinting^26–28^. In this context, Hyperspectral Imaging (HSI) has emerged as a powerful set of techniques that integrate conventional imaging and spectroscopy^29, 30^. Unlike a typical micrograph, a hyperspectral image provides a complete spectrum of the sample at every pixel location, so that it is possible to gather morphological features together with local spectral information. Among available HSI methods, Fourier Transform InfraRed (FT-IR) imaging^31, 32^ and Raman microspectroscopy^33, 34^ represent two of the most promising tools for bioanalytical and biomedical sciences. In particular, Raman microspectroscopy has benefited from technological advances that have enabled biological samples to be analysed with improved signal or more suitable measurement speed^35^. These include Resonance Raman Scattering (RRS)^36^, Surface-Enhanced Raman Scattering (SERS)^37^, and Coherent Anti-Stokes Raman Scattering (CARS)^38, 39^. A comprehensive overview of the advances in nonlinear Raman microspectroscopy con be found in the recent literature^40^.

Refractive index represents another interesting target to monitor crucial changes inside living cells^41^ without the use of exogenous markers that could affect cell behaviour. Variations of refractive index as a result of cell rearrangement in response to stimuli have indeed an effect on the visible and near infrared reflectance spectrum of the cell. The introduction of reflectance HSI as a specific feature of Laser Scanning Confocal Microscopy (LSCM) following the development of white laser sources is quite recent and not common^42^.

Here, we describe the use of a hyperspectral confocal microscope aimed at integrating structural and morphological information with detailed spatially resolved spectroscopic properties, powered by a supercontinuum laser source in the visible and near infrared spectral ranges, and able to collect a complete spectroscopic image by the acquisition of a one-shot wide range reflectance spectrum for every image pixel in the three-dimensional dataset typical of a confocal microscope^43^.

Label-free reflectance HSI has already been proven as a tool for discriminating cellular compartments^43^, cells at different stages of apoptosis^44^ and different cell types in an *in vitro* co-culture model^45^. In the present work, we detail the application of HSI to the *in vitro* label-free characterization of classically (M1) and alternatively (M2) polarized monocyte-derived macrophages (MDM).

## Methods

### Cell isolation and differentiation

MDM differentiation and polarization were performed slightly modifying the protocol described by Mantovani *et al*.^46^. Buffy coats from male healthy donors were obtained at the Blood Donor Centre at Policlinico Universitario Campus Bio-Medico after written informed consent, according to the institutional guidelines. Human peripheral blood mononuclear cells (PBMCs) were isolated from buffy coats by Lympholyte®-H density gradient (Cedarlane Laboratories). Monocytes were sorted by magnetic activated cell sorting (MACS) using magnetic beads conjugated with anti-human CD14 (Miltenyi Biotech) and cultured for 6 days in RPMI 1640 culture medium (Euroclone) supplemented with 10% foetal bovine serum (Hyclone, Thermo Fisher Scientific), 5% human serum (Sigma-Aldrich), 100 units/mL penicillin, 100 mg/mL streptomycin (Euroclone), 2 mM l-glutamine (Euroclone) and 25 ng/mL colony stimulating factor 1 (CSF-1) (alias Macrophage CSF, M-CSF, PeproTech) to differentiate them into non-polarized (Mϕ) MDM. M1 polarization was achieved by supplementation with interferon-γ (IFN-γ, 10 ng/mL, PeproTech) and lipopolysaccharides from *E*. *Coli* (LPS, 100 ng/mL, Sigma-Aldrich) for 48 hours, whereas M2 polarization was obtained by supplementing cells with interleukin-4 (IL-4, 20 ng/mL, PeproTech) for 48 hours. After polarization, M1 and M2 populations were split into different subsamples to perform molecular, flow cytometry and spectroscopic characterizations.

### Fluorescence Imaging

MDMs were fixed in 4% PFA for 10 min and permeabilised with 0.1% Triton X-100 (Sigma-Aldrich). After washing, cytoskeletal actin was labelled with FITC-phalloidin (Sigma-Aldrich, 1:400 in PBS for 45 min), and nuclei were counterstained with DAPI (Thermo Fisher Scientific, 1:10000 in PBS for 15 min).

### Flow cytometry

MDMs were stained with monoclonal mouse anti-human CD68, CD80, CD86, CD163, and CD206 (mannose receptor, MR) antibodies (eBioscience, Affymetrix). Cells were suspended in PBS at a concentration of 1 × 10^5^ cells/mL. Non-specific antigens were blocked by 5% BSA buffer. For intracellular CD68, fixation in 4% PFA and permeabilisation with 0.1% Triton X-100 were achieved prior to staining. Cells were analysed using a BD FACSCanto II flow cytometer (BD Biosciences). Non-specific mouse Igs were used as an isotype control.

### Gene expression

Gene expression levels were evaluated by two-step Quantitative Reverse Transcription PCR (RT-qPCR). Isolation and purification of mRNA were performed using TRI Reagent (Sigma-Aldrich). Extracted mRNA was quantified by spectrophotometric technique (Nanodrop, Thermo Fisher Scientific). 1 μg of total RNA was reverse-transcribed using High Capacity cDNA Reverse Transcription Kit (Life Technologies) according to the manufacturer’s instructions. Amplification was performed on 50 ng of cDNA in a total reaction volume of 20 μL, using TaqMan Universal MasterMix II (Life Technologies) and primers (TaqMan Gene Expression Assay, Life Technologies) for the following genes: interleukin-10 (IL10, Hs00961622_m1), interleukin-12 (IL12A, Hs00168405_m1), arginase (ARG1, Hs00968979_m1), inducible nitric oxide synthetase (NOS2, Hs01075529_m1), tumour necrosis factor-α (TNFA, Hs01113624_g1), and CD206 (MRC1, Hs00267207_m1). Beta-actin (ACTB, Hs01060665_g1), glyceraldehyde 3-phosphate dehydrogenase (GAPDH, Hs02758991_g1) and cyclophilin A (PPIA, Hs04194521_s1) were used as a set of endogenous controls (following their validation with Bestkeeper software^47^).

### Statistical analysis

Statistical analysis of flow cytometric and gene expression data was performed using GraphPad Prism ver. 6.0 suite (GraphPad Software). Conditions of normality were checked and met. Student’s t-test was used for means comparison between M1- and M2-polarised cells. Significance was set at the 0.05 level.

### HSI and multivariate analysis

After polarization induction, MDMs were transferred to dedicated culture chambers^43^, composed of a circular glass coverslip (170 μm thickness, 40 mm diameter), surmounted by a 2-mm-thick plasma-bonded annular polydimethylsiloxane gasket. After overnight incubation, chambers were closed with a glass coverslip lid and mounted on a sample holder which was then fixed on the microscope stage.

Specimens were observed under a custom built hyperspectral confocal reflectance microscope, equipped with a supercontinuum white light laser (SuperK, NKT Photonics). Details on the microscope design and optical performance are provided elsewhere^42, 43^.

The hyperspectral microscope was equipped with a 36 × 0.52 N.A. reflective objective. Square ROIs (1 × 1 mm^2^ wide) were recorded with a 512 × 512 pixel resolution at 400 Hz. The spectral resolution was 1.6 nm in the wavelength range considered for elaboration (500–1000 nm). Illumination dose was tested against possible photo-damage effects. Image analysis protocol included 30 randomly chosen cells per field of view. For each cell, an averaged spectrum on a circular region (12 pixel diameter, equivalent to 23.4 µm) centred in the nuclear area, was extracted from the hyperspectral images using a custom software. Spectra were then analysed using Origin ver. 9.1 suite (OriginLab).

Cell spectra were treated for background subtraction using signal from free adjacent areas and further smoothed using a Savitzky-Golay algorithm. Principal Component Analysis (PCA) was used to analyse spectra, considering the intensities over 26 discrete equidistant wavelengths ranging from 500 to 1000 nm (20 nm spacing).

PCA is a multivariate statistical method for the visualization of the information contained in a data matrix to reduce the original data complexity. This result is obtained by a transformation from the n-dimensional space of *n* original variables (26 sampling wavelengths) into a space in which the new variables (principal components) and hence dimensions are ordered hierarchically according to the variance of data points. In this way, considering only a limited number of dimensions has a limited effect in terms of loss of information.

From the analysis of the loading plot (*i*.*e*., the principal component coefficients in the sample space) for the principal component along which the M1/M2 separation occurred, the spectral regions involved in the characterization of the two cell types were identified. A set of the most representative wavelengths was used for Linear Discriminant Analysis (LDA), a trained statistical algorithm for feature reduction and object classification that was implemented in MATLAB (rel. 2015b, The MathWorks).

The dataset consisted in 60 observations (30 M1 and 30 M2) for each donor. Donors were first analysed individually, and a stratified 10-fold cross validation method^48^ was chosen to recursively train-test the LDA model. Then, the whole dataset comprising the observations from all donors was considered and subsampled for training-test using a stratified 10-fold cross validation method.

In both cases, confusion matrices and classification errors were calculated as the summation of values from the 10-fold procedure.

## Results

### Assessment of Macrophagic Polarization

**Figure 1:**
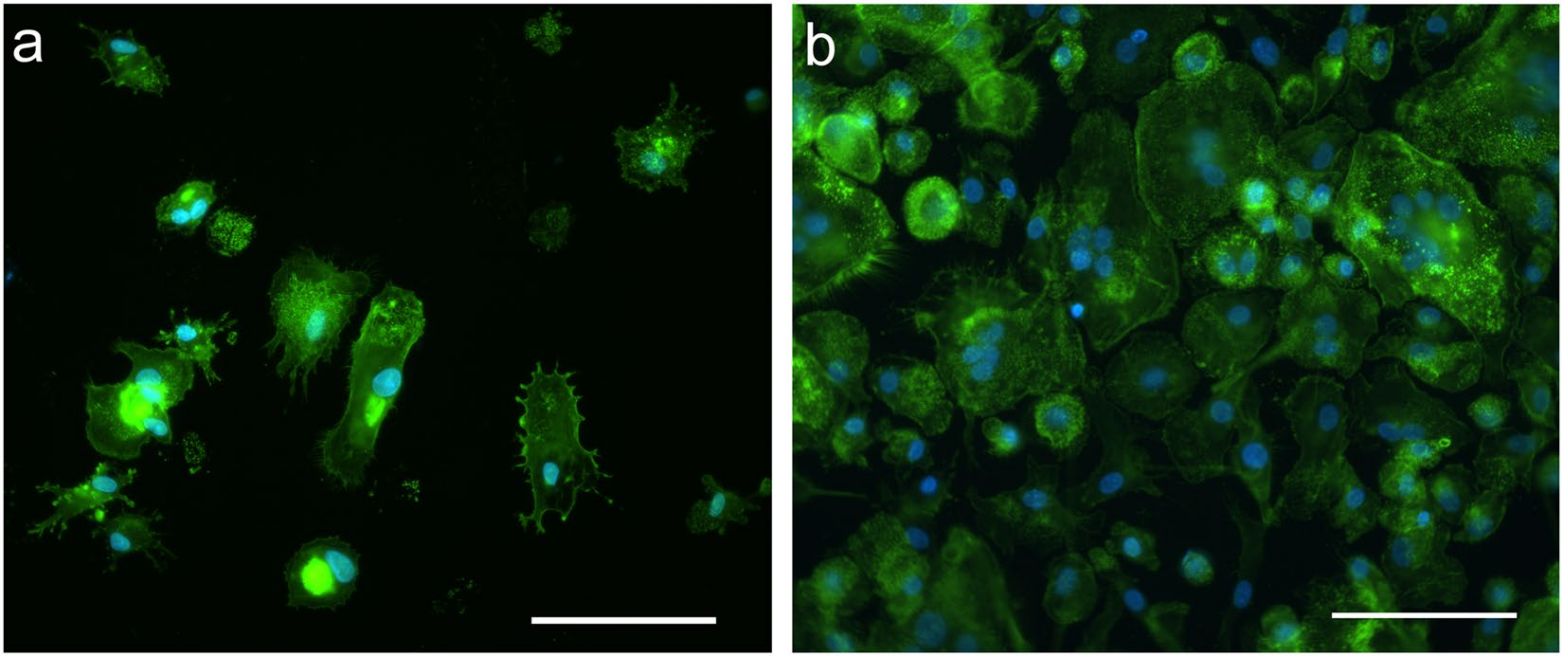
Epifluorescence microscopy. M1 (**a**) and M2 (**b**) MDMs labelled with FITC-phalloidin to stain cytoskeletal actin (in green), and DAPI nuclear counterstain (in blue). Scale bar: 100 μm.

Following MDM differentiation and polarization, the two resulting cell populations showed distinct morphological features. Figure 1 shows the morphology of M1 and M2 MDM cells as observed by epifluorescence microscopy. M1 MDMs showed a spindle shaped morphology, while M2 MDMs exhibited a more spread morphology, with the presence of giant multinucleated cells.

**Figure 2:**
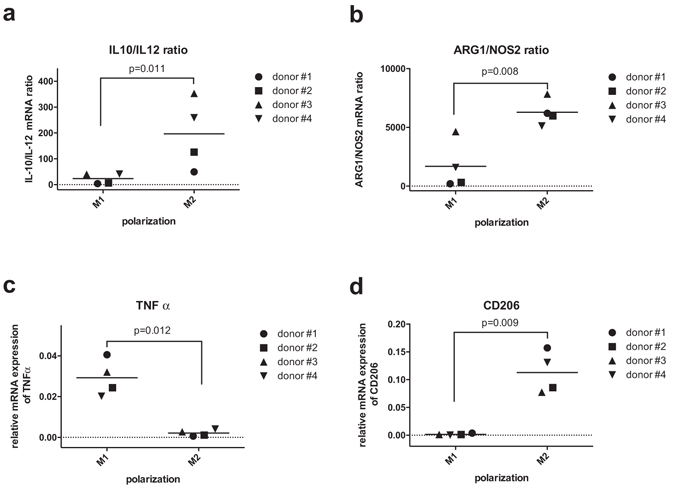
Gene expression profile evaluated by RT-qPCR. Scatter plots of IL-10/IL-12 mRNA ratio (**a**), ARG1/NOS2 mRNA ratio (**b**), TNF-α relative mRNA expression (**c**) and CD206 relative mRNA expression (**d**) are reported.

Macrophage M1/M2 polarization was also evaluated by gene expression and flow cytometry assays. Figure 2 reports the results of gene expression assays performed by RT-qPCR. For each donor, M1 MDMs showed lower IL-10/IL-12 (Fig. 2a) and arginase/iNOS (Fig. 2b) ratios compared to M2 ones. Although a great variability among donors has to be evidenced, the differences between M1 and M2 mean values were statistically significant and in substantial accordance with previous literature^49^. M1 macrophages also showed significantly higher TNF-α (Fig. 2c) and significantly lower CD206 (Fig. 2d) expression levels compared to M2 ones.

**Figure 3:**
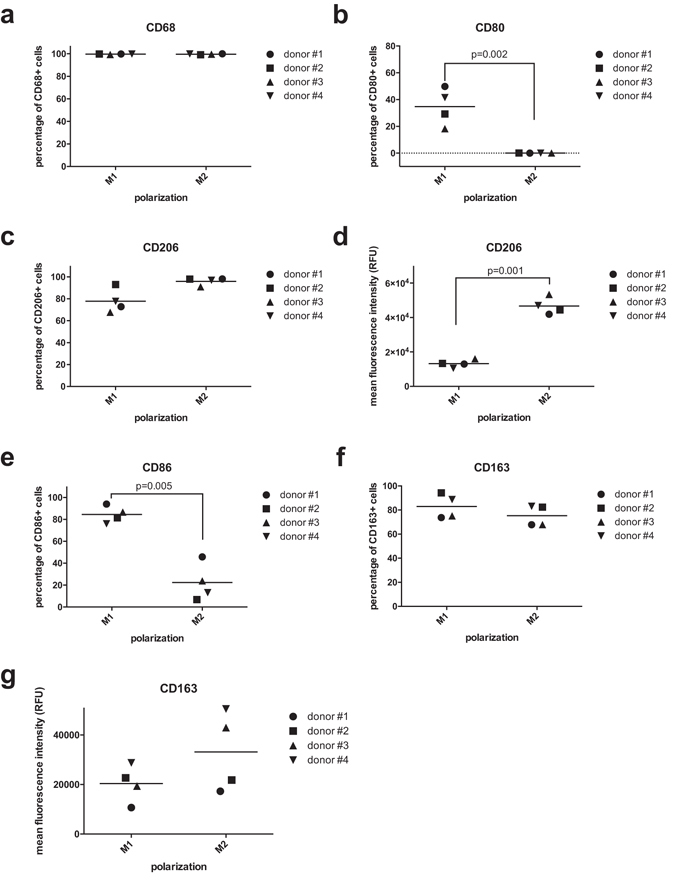
Flow cytometry. (**a**–**c**) Positivity to pan-macrophagic marker CD68 (**a**), M1 marker CD80 (**b**) and M2 marker CD206 (**c**). (**d**) Mean fluorescence intensity levels for CD206. (**e**,**f**) Positivity to M1 marker CD86 (**e**) and M2 marker CD163 (**f**). (**g**) Mean fluorescence intensity levels for CD163.

The results of flow cytometric characterization are reported in Fig. 3. Pan-macrophagic CD68 marker was ubiquitously expressed both in M1 and in M2 groups (Fig. 3a). M1 were CD80^+^ CD206^low^, while M2 macrophages showed a CD80^−^ CD206^high^ profile (Fig. 3b–d).

Consistently with literature findings, CD86 was highly expressed in M1 cells (Fig. 3e), while CD163, that is commonly associated to M2 polarization^50^ (although it may not be considered as a conclusive M2 marker^51^), was not successful in discriminating the two groups with statistical significance (Fig. 3f,g).

### HSI and Principal Component Analyjsis

**Figure 4:**
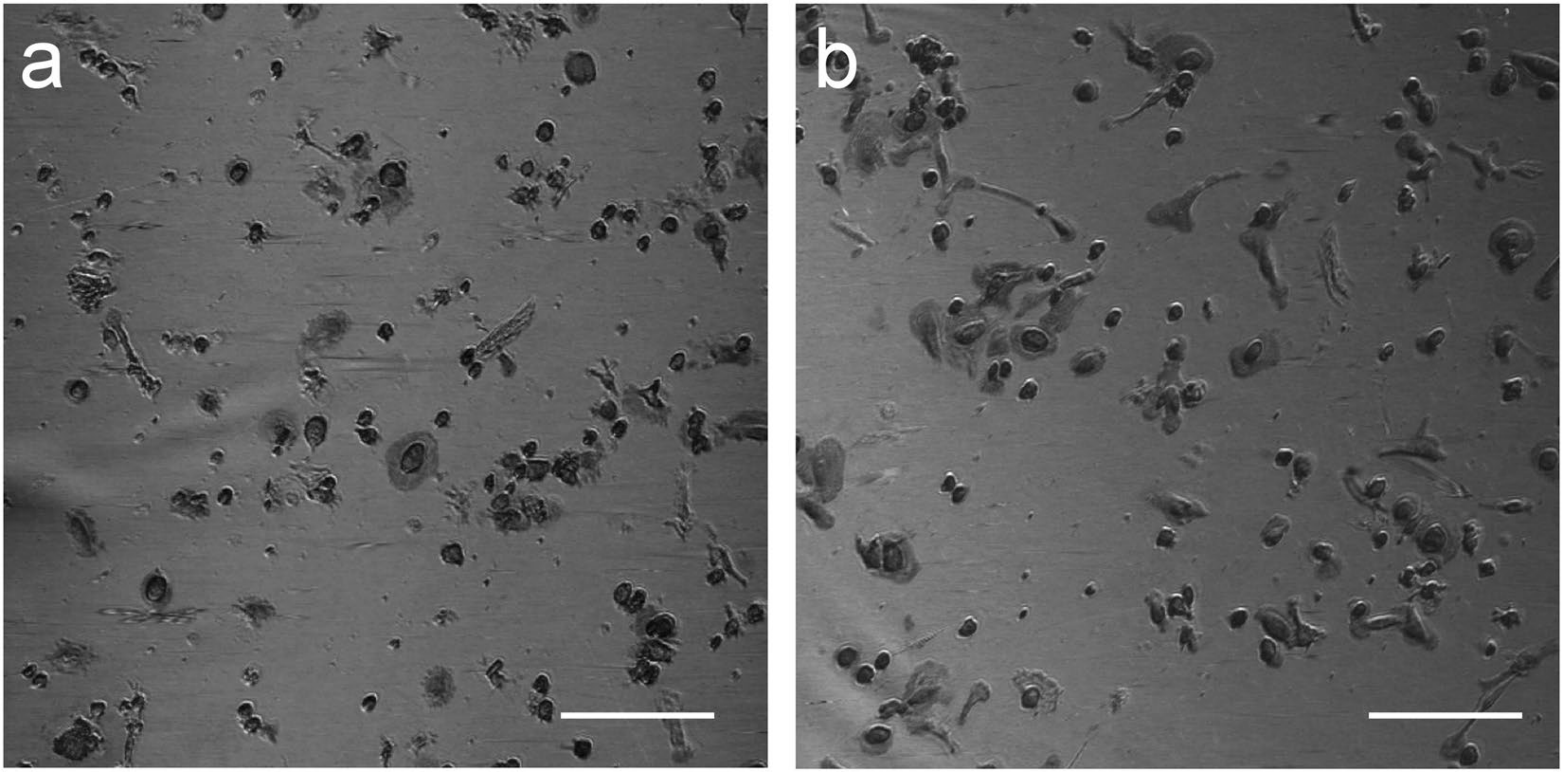
Hyperspectral microscopy. Representative micrographs for M1 (**a**) and M2 (**b**) MDMs obtained as a grey level representation of the reflectance value at 700 nm, as extracted from the hyperspectral datasets. Scale bar: 200 μm.

A panel of typical HSI micrographs for M1 and M2 MDMs (for the representative wavelength of 700 nm) is reported in Fig. 4, while HSI lambda stacks for M1 and M2 MDMs are available as Supplementary Videos SV1 and SV2, showing the wavelength dependence of the reflectance intensities (montage was performed using Fiji^52^). Typical cell spectra extracted from HSI datasets are also provided in Supplementary Figure S1.

**Figure 5:**
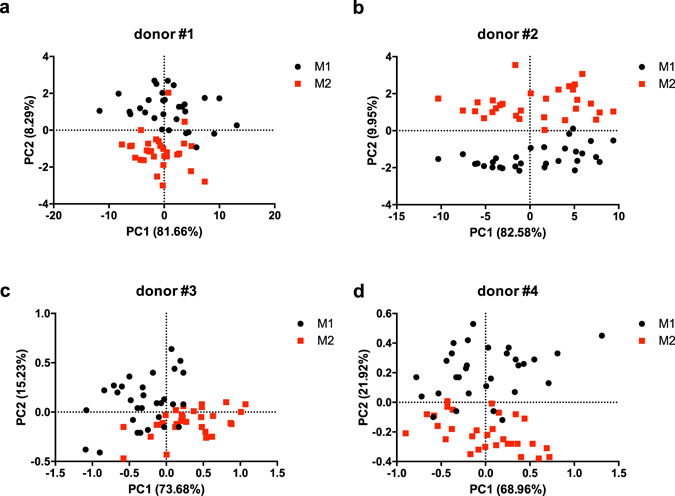
Principal Component Analysis. PCA score plots of MDM spectra for different donors. Spectra of M1 and M2 MDMs are represented as black circles and red squares, respectively.

Figure 5 shows the PCA score plots obtained for the different donors. For each donor, spectra from M1 and M2 MDMs have been considered together to define the Principal Component space (further details are provided as Supplementary Figures S2 and S3). As for the results of biological characterization, a high donor-to-donor variability was reported; nonetheless, from the analysis of PCA score plots, a clear separation between M1 and M2 groups along PC2 direction could observed. Indeed, PC2 accounted for 8 to 22% of the total variance, and a classification based on the position of points in the positive or negative PC2 half-plane led to a mean error in the order of 10%.

### Linear discriminant analysis

**Figure 6:**
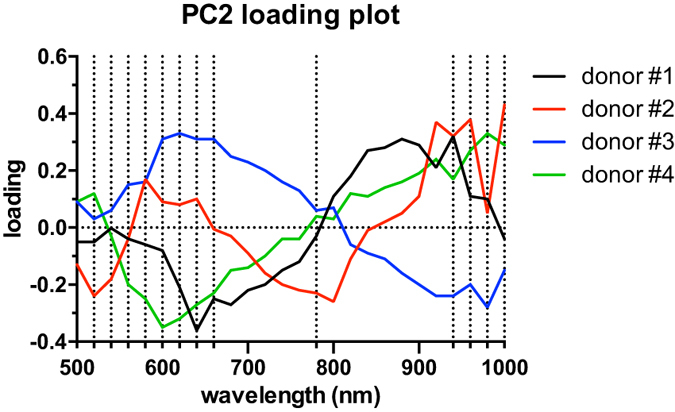
PC2 loading plot. Loading plot for the second principal component (PC2), along which the M1/M2 separation occurred. Wavelengths chosen for LDA analysis are marked by dotted lines.

After identifying PC2 as the direction along which the separation between cellular spectra took place, the loading plot of PC2 for the different donors was considered (Fig. 6). Fourteen wavelengths were then chosen as a set of predictors for LDA-based automatic classification, privileging the spectral ranges characterized by high loading coefficients (marked by dotted lines in Fig. 6).

**Figure 7:**
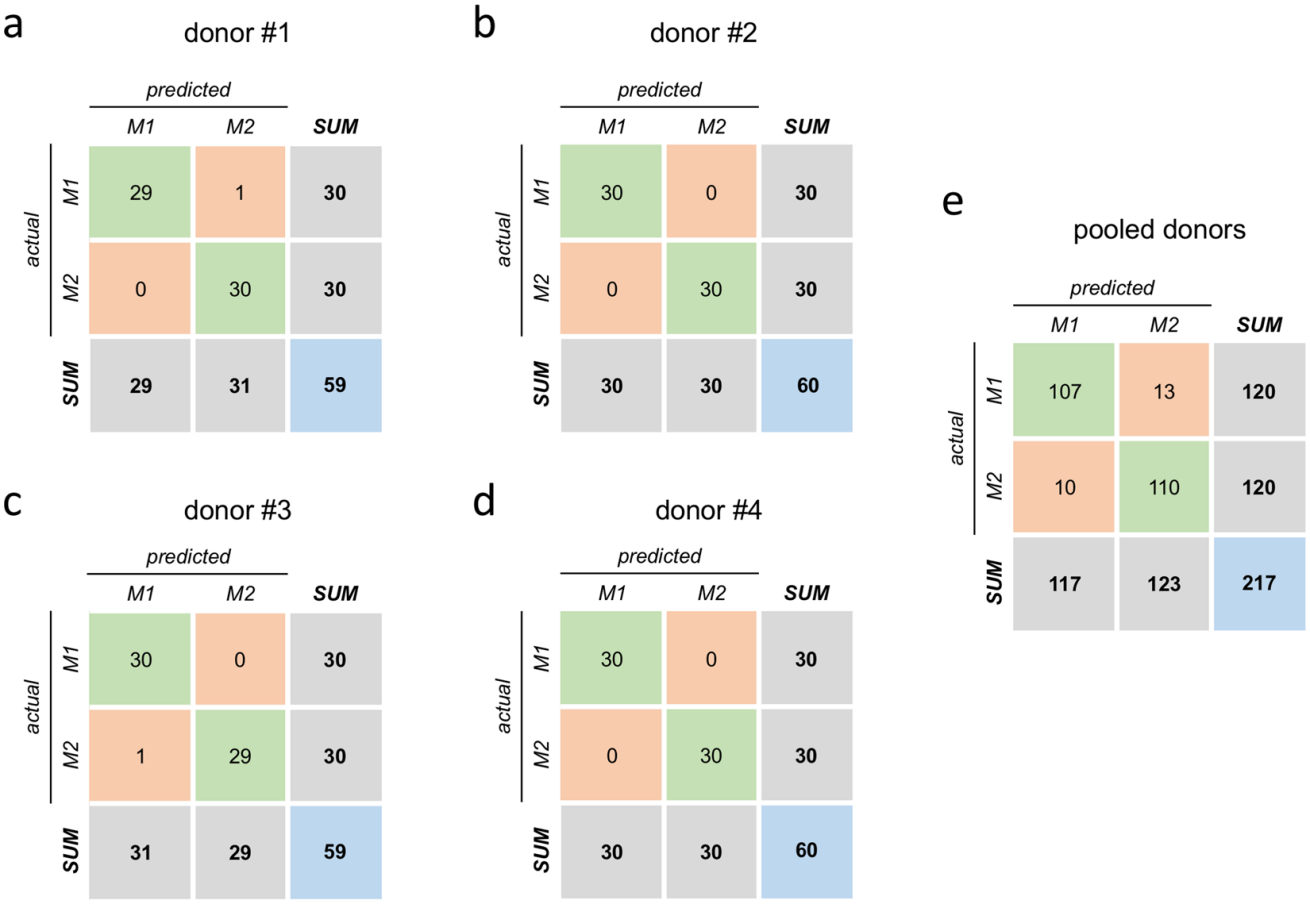
LDA model. Confusion matrices from 10-fold cross validation (each matrix is the summation of 10 confusion matrices from 10 test sets).

When donors were analysed individually, classification accuracy ranged between 98% and 100% (Fig. 7a–d). The possibility to define more general classification features has been explored by pooling together observations from different donors. This latter approach was affected by a higher error (classification accuracy above 90%, Fig. 7e) than the intra-individual one.

## Discussion

The use of spectroscopic techniques for diagnostic decision support has gained wide attention in the recent years as a promising tool toward precision medicine^53^. This is particularly true regarding macrophagic polarization in cancer, as per the documented correlation of TAM infiltration as a negative prognostic factor in the progression of the disease. Conversely from other literature works that have tackled macrophagic polarization using spectroscopic tools addressing specific light-matter interactions (*e*.*g*., fluorescence lifetime imaging microscopy^26, 27^, forward angular light scattering^28^) for the establishment of decision support systems based on chemometry, the proposed approach exploits a simpler physical phenomenon, namely Vis-NIR reflectance^54^, for the analysis of cells *in vitro*. As a major advantage, our system has the potential for easy integration into routine equipment (microscopes), or even into cytometric setups, and is in principle compatible with other wide-spectrum light sources, such as cost-effective metal halide lamps.

Consistently with data reported in the literature, PBMC collected from healthy donors were successfully differentiated into macrophages (MDM) showing either M1 or M2 polarization.

In this regard, reference has to be made to the recent work of Murray and colleagues^55^, who posed the accent on the need for improved nomenclature and standardization in research on macrophages, as a *lingua franca* has yet to be established and accepted. The Authors established a set of standards encompassing three principles: source of macrophages, definition of the activators, and a consensus collection of markers to describe macrophage activation, with the goal of unifying experimental standards for diverse experimental scenarios. Additionally, the authors proposed a novel cell nomenclature which unambiguously defines the differentiation protocol underlying the cells under investigation. In line with this view, the present study discloses with proper detail cell source and differentiation protocols. For the sake of simplicity, however, the M1/M2 nomenclature was retained in the present manuscript, acknowledging the correspondent M(IFN-γ/LPS) and M(IL-4) classification.

Additionally, when analysing *in vitro* differentiated MDMs, it has to be clearly acknowledged that the M1 vs. M2 paradigm represents an *in vitro* extremization of the *in vivo* setting, in which a “continuum” of activation states exists^56^.

In light of the results of gene expression and surface marker characterization, a high donor-to-donor variability in terms of surface markers and mRNA levels has to be reported, as also documented elsewhere^57^; nevertheless, the set of molecular markers presented in this work helps to identify M1 and M2 class with good reliability.

Such a biological donor-to-donor variability was mirrored by the results of PCA analysis on hyperspectral datasets. Indeed, when pooling together several donors (as reported in Supplementary Figure S4 for the representative case of donors #3 and #4), PCA failed to provide a clear separation between M1 and M2 populations. The same result was confirmed by LDA analysis: classification accuracy was very high when training and test sets were limited to a single donor, while the pooled dataset was affected by a larger error.

The significance of this work relies on the possibility to assess macrophagic polarization *in vitro* using a label-free live-cell imaging methodology. The proposed approach has the potential to be integrated within a wide spectrum of experimental designs, including co-cultures with tumour cells to study the tumour/immune interface^58, 59^, with the possibility to detail the kinetics of polarization shift at the single cell level, paving the way to personalized drug testing and therapy assessment. In such a scenario, the reported restriction to a single PBMC donor for improved classification efficiency shall not be retained as a major drawback.

## Electronic supplementary material

Electronic Supplementary Information

Video SV1. Pseudo-coloured lambda stack for M1 MDMs.

Video SV2. Pseudo-coloured lambda stack for M2 MDMs.
