# Supplementary material for: Classification of M1/M2-polarized human macrophages by label-free hyperspectral reflectance confocal microscopy and multivariate analysis
Source: Sci Rep. 2017 Aug 21;7:8965. doi: 10.1038/s41598-017-08121-8 (PMC5566322; doi:10.1038/s41598-017-08121-8)
Supplement: Supplementary file 1 — Electronic Supplementary Information [file 41598_2017_8121_MOESM1_ESM.pdf]

ELECTRONIC SUPPLEMENTARY INFORMATION

Classification of M1/M2-polarized human macrophages by label-free hyperspectral reflectance confocal microscopy and multivariate analysis

Francesca R. Bertani, Pamela Mozetic, Marco Fioramonti, Michele Iuliani, Giulia Ribelli, Francesco Pantano, Daniele Santini, Giuseppe Tonini, Marcella Trombetta, Luca Businaro, Stefano Selci, Alberto Rainer

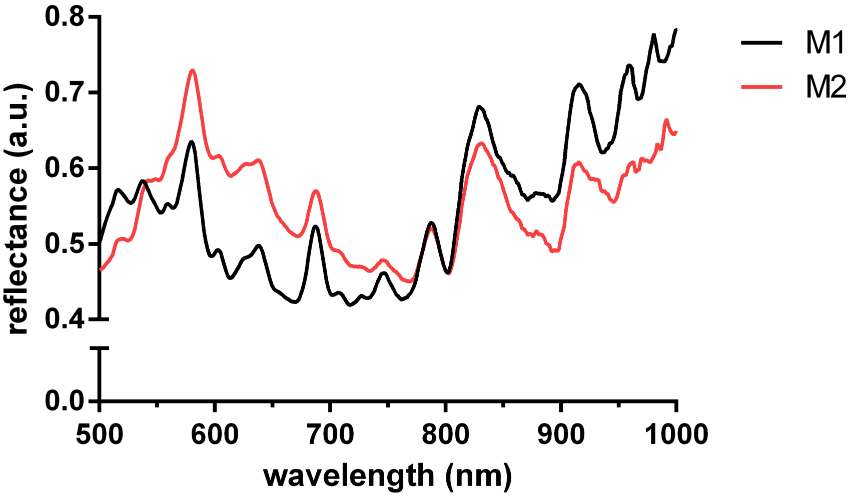

Figure S1. Reflectance spectra. Typical reflectance spectra extracted from the HSI datasets for the representative case of donor #4. Each spectrum represents the average signal from a 12 pixel circular ROI centered on the cell nucleus, following background subtraction and Savitzky-Golay filtering.

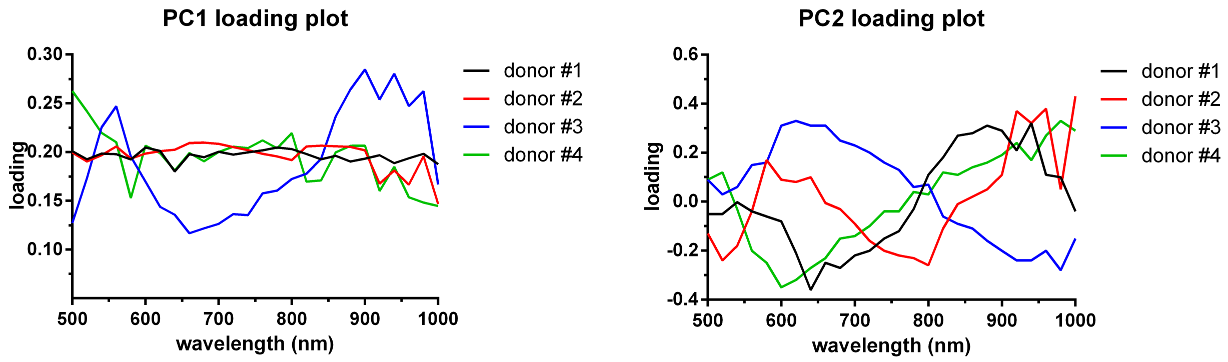

Figure S2. Loading plots. Loading plots of the two first principal components (PC1 and PC2) for different donors.

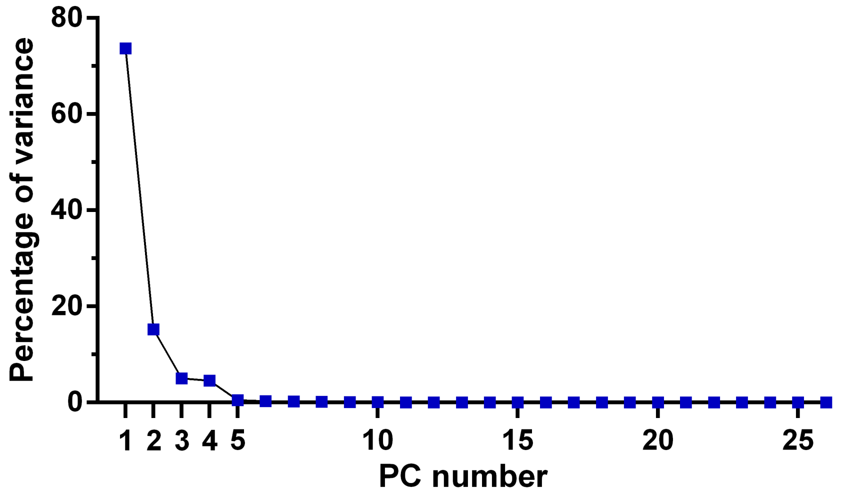

Figure S3. Scree plot. Percentage of variance displayed by the different principal components for the representative case of donor #3.

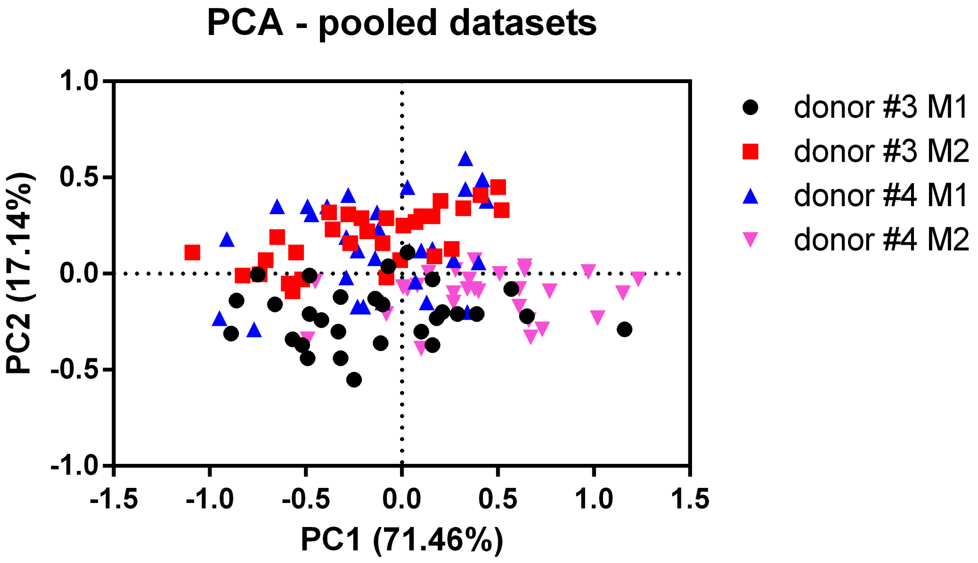

Figure S4. PCA for pooled datasets. PCA score plot of MDM spectra from two different donors. When performed on pooled datasets, PCA fails to provide clear separation along either PC1 or PC2.

Video SV1. Pseudo-coloured lambda stack of a HSI dataset for M1 MDMs. Scale bar: 200 µm.

Video SV2. Pseudo-coloured lambda stack of a HSI dataset for M2 MDMs. Scale bar: 200 µm.
